# Distribution of *Trichostrongylus colubriformis* on common pasture grasses and legumes from the Midwestern United States

**DOI:** 10.1371/journal.pone.0321367

**Published:** 2025-04-11

**Authors:** Katy A. Martin, Jeba R.J. Jesudoss Chelladurai, Christina Lavery, Rachel Shelangoski, Matthew Chaffee, Matthew T. Brewer

**Affiliations:** 1 Department of Veterinary Pathology, College of Veterinary Medicine, Iowa State University, Ames, Iowa, United States of America; 2 Department of Pathobiology, College of Veterinary Medicine, Auburn University, Auburn, Alabama, United States of America; Beni Suef University Faculty of Veterinary Medicine, EGYPT

## Abstract

Nematodes that infect grazing herbivores rely on the ability of larvae to escape the fecal pat and ascend onto forage in order to be eaten by a subsequent host. However, pastures are polycultures of grasses and forbs that vary with respect to morphology and potential innate defense mechanisms acquired as part of their own co-evolution with nematode parasites. The objectives of this study were to 1) characterize the vertical distribution of *Trichostrongylus colubriformis* on a variety of plant species found in pastures in the Midwestern United States and 2) to identify plants that enhanced or inhibited larval ascent. Climbing assays were performed under greenhouse conditions whereby L3 were directly recovered from foliage. We found that at least 50% or more of the larvae were distributed on the first 2.5 cm closest to the soil surface for all plant species tested. In contrast, less than 10% of the larvae were distributed 12 cm or higher. For practical purposes, our findings agree with previous studies that suggest limiting grazing below a certain height to decrease parasite consumption. Further nuanced studies are needed to identify individual plant mechanical and chemical defenses that impact the ecology nematodes of veterinary importance.

## 1. Introduction

Strongyle nematodes are common gastrointestinal parasites of herbivores including farmed ruminants such as sheep and cattle. Parasite eggs are shed in feces, where an L1 larva develops and hatches from the egg, molts, and ascends onto forage where it is more likely to be eaten by a subsequent host [1]. There has been a massive effort utilizing anthelmintic agents to mitigate losses associated with nematodes. Conventionally, the approach to nematode control in farmed ruminants involves observing egg shedding in the host and attempting to avoid clinical disease with the use of anthelmintics [2]. On the other hand, strategies for control of larva on pasture have been developed but are not easily deployed in most grazing systems [3]. Broadly, we hypothesize that the utilization of plant- and forb-based biocontrol is one way to control of strongyle larvae in the pasture environment.

Many studies have investigated survival of strongyles from the standpoint of environmental conditions. Examining the effect of weather, climate, or microconditions on the number of L3 available to infect grazing animals has led to models predicting parasite survivability [4,5]. These studies have largely considered environmental abiotic conditions but less so on biotic factors such as plants or other organisms in the landscape [4,6,7]. Vegetative plants including grasses and legumes, for example, have developed cuticular waxes, glandular trichomes, and chemical effectors to deter plant pathogenic nematodes [8] and herbivorous insects or vertebrates [9]. We hypothesize that strongyle parasites, although they are bystanders in the plant-plant pathogen context, may succumb to innate plate defenses thereby suggesting that some plant species could be deployed to decrease strongyle larvae in the landscape.

Since the L3 rely on the ability to ascend forage and be eaten, they must successfully navigate anti-nematode defense mechanisms on the surfaces of plants. In addition, grasses and legumes vary with respect to morphology of structures such as leaves, stems, trichomes, etc. The structural morphology of pasture forage plant species varies substantially which may hinder or assist nematodes during their ascent. In this study we selected *Trichostrongylus colubriformis*, a strongyle with a worldwide distribution, as a model for studying trichostrongyle interaction with pasture forage. Using a direct test of climbing ability, we measured the ability of L3 to ascend different pasture forage species that would impact practical grazing recommendations. Secondarily, we aimed to indirectly test the hypothesis that the physical and/or chemical defenses of different pasture forage species would lead to variation in the distribution of strongyle larvae on the plants.

## 2. Methods

### 2.1. Parasites and plants

*Trichostrongylus colubriformis* strains were obtained opportunistically from naturally infected animals. Following identification, parasites were amplified in calves to generate L3 cultures that were > 95% pure *T. colubriformis*. Local laws, IACUC compliance were verified by the vendor (Myers Parasitology Service, Magnolia, KY). Coproculture and standard Baermann sedimentation were used to concentrate and wash larvae. After collection, larvae were used in experiments within two weeks. Pasture forages were obtained from a commercial source (Triple A Seeds, Carroll, Iowa, USA). Forages (with scientific and local colloquial name provided) included *Melilotus officinalis* (sweet clover), *Trifolium pratense* (red clover), *Medicago sativa* (alfalfa), *Phleum pretense* (timothy), *Sorgham* spp (sudangrass), *Lolium perenne* (perrenial rye), *Dactylis glomerate* (orchard grass), *Poa pratensis* (Kentucky bluegrass), *Sorghastrum nutans* (indiangrass), and *Bromus inermis (*smooth brome).

### 2.2. Forage grown under greenhouse conditions

Seeds were planted in pots containing a mixture of commercial topsoil and potting soil (Miracle Grow commercial potting mix) and grown in a green house on the central campus of Iowa State University in Ames, Iowa, USA (42.0267° N, 93.6465° W). Seeds were distributed in soil in typical nursery plastic pots (Lowe’s Companies, Inc #612821). Pots were watered daily on an arbitrary schedule. Greenhouse conditions were maintained and plants watered on an arbitrary daily schedule. Monitored conditions in the greenhouse included temperate (22–25°C) and humidity (60–65%). Plants were subject to ambient light conditions (translucent greenhouse roof), with no manipulation cycles during the months of June and July.

### 2.3. Larval climbing assays

Experiments were conducted a minimum of three times in triplicate whereby each biological replicate consisted of a 3 L container of soil, hand planted with each plant species. Technical replicates were created in each plant pot by randomly inserting a plastic cup with the bottom cut off to denote an area of 78.5 cm^2^ for each replicate. Using a serological pipette, 1000 *T. colubriformis* L3s were transferred to the base of the grass plants in this study area such that the cup would restrict the larvae from escaping to other areas of the pot. Parasites were allowed to ascend the plant for 48 hrs. Segments of the plants (~2 cm each) were harvested using a scissors from the top of the plant downward until the soil was reached.

### 2.4. Enumeration of larvae from forage

Each sample of foliage was placed in a 50 mL conical tube containing room temperature tap water. The slurry including plants and water vortexed and placed in a Baermann apparatus for 24 hrs in order to concentrate the L3 into a concentrate that was 5 mL or smaller. Next, the concentrated slurry was passed through a 5 µm polycarbonate filter that was placed on a gridded microscope slide and the total number of larvae was counted by the same individuals (CL and MC) for each biological replicate.

In preliminary studies, the standard Baermann procedure did not produce a concentrated adequate numbers of L3 sufficient for counting. Thus, we adapted the “difil-test” designed to filter microfilarial nematodes from blood [10]. Using a syringe, we forced forage rinsates through 0.2 µm membranes screwed on to the luer lock hub of the syringe. After membrane capture, the L3 could be more easily and efficiently enumerated. Preliminary experiments demonstrated relatively little difference between 24 and 48 hrs, so we selected 48 hrs as the experimental timepoint. The kinetics of movement beyond 48 hours under different climactic conditions requires further investigation.

### 2.5. Data analysis

The total number of larvae recovered were used to calculate the percentage of parasites that ascended on to the forage after 48 hours. Counts were used to calculate the percentage of recovered larvae present from each plant species which was analyzed by one-way ANOVA. The relative percent of larvae recovered from each spatial zone was analyzed by two-way ANOVA using the forage species as the column affect and the spatial zone as the row effect. Tukey post-hoc test was used to determine if the mean percentage of larvae differed. Results were analyzed and visualized with Graphpad Prism 9.

## 3. Results

### 3.1. Only a proportion of larvae ascend forage

**Fig 1:**
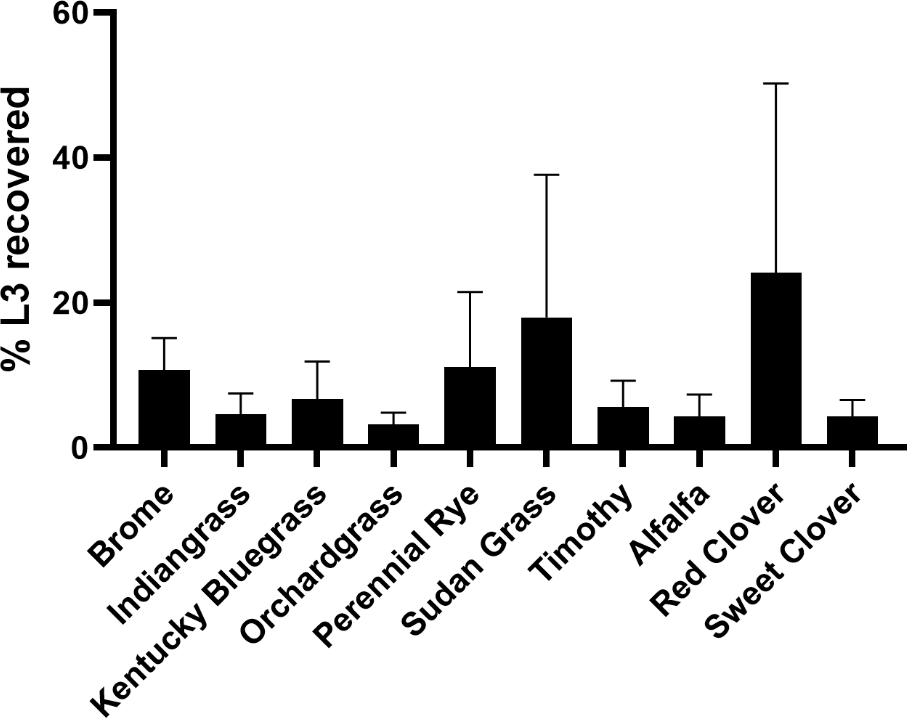
Proportion of L3 recovered from forages. Percent of total number of L3 recovered from total plant biomass relative to the initial number of parasites placed at soil level in test plots. Bars represent mean ±  SD of three independent experiments. One-way ANOVA did not detect significant differences (Plant species accounting for the difference among means p = 0.38).

In general, our experiments found that less than 10% of the total number of L3 ascend onto the forage within 48 hours. Red Clover and Sudan grass appeared visually to have a higher recovery rate of around 20% (Fig 1). However, these differences were not statistically significant. We attributed this to low recovery rates from red clover and sudan grass in a single replicate experiment, possibly from plant biomass drying out under ambient conditions.

### 3.2. The majority of larvae are distributed on the first 2.5 cm above the soil surface

**Fig 2:**
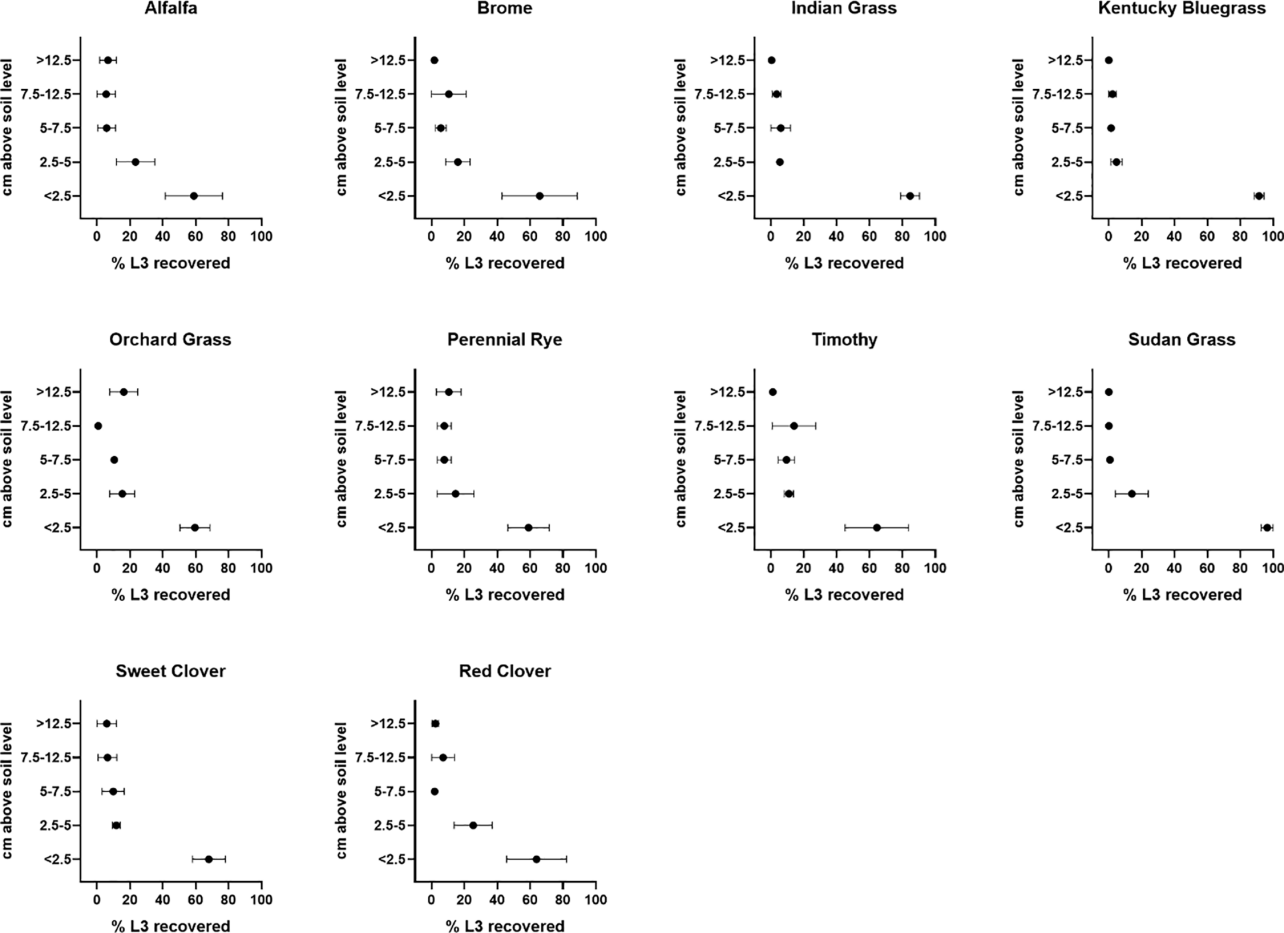
Distribution of total *T. colubriformis* recovered after 48 hours, stratified by height zone. Values represent the proportion of larvae in each zone relative to the total number of larvae recovered from each forage species. Dots represent the mean of 3 technical replicates for each of 3 independent experiments ±  SEM. The percent of larvae found between soil level and 2.5 cm was significant compared to other spatial zones within each plant species (Complete post-hoc testing data provided in Table 1).

**Fig 3:**
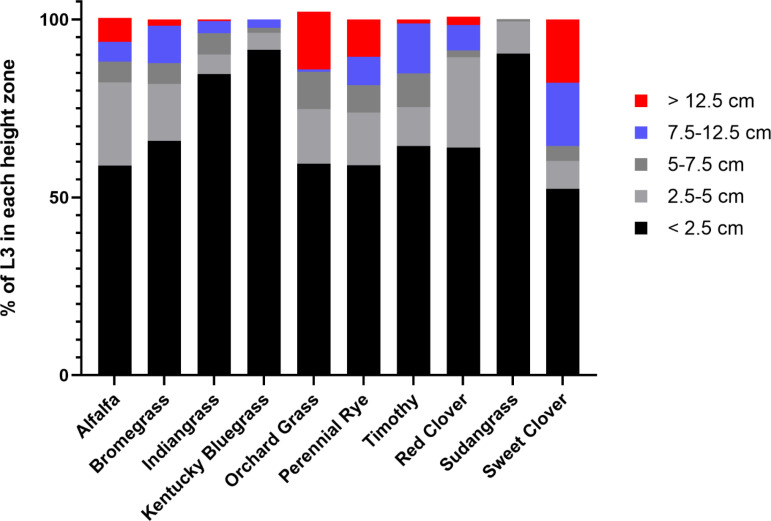
Parts of a whole analysis visualizing the relative distribution of *T. colubriformis* L3 in each height zone.

Few larvae were found on the relatively higher parts of the plant. For all plants investigated, greater than 50% of the total recovered L3 were recovered from the lowest 2.5 cm of the plant above the soil surface (Figs 2 and 3). In contrast, a few larvae were found more than 12.5 cm above the soil surface and this consisted of 1–10% of the total larvae recovered depending on the plant species (Fig 2). Two-way ANOVA revealed that the 0–2.5 cm spatial zone was different from all other spatial zones for the forage species tested. Values for post-hoc testing for multiple comparisons spatial zone within a forage species are show in Table 1. Analysis did not detect differences within zones of different species; the corresponding confidence intervals showing this data are given in supplemental S1 Table.

**Table 1:** Differences among recovery of larvae from different heights above soil level. Tukey’s multiple comparison test; height above soil level are given for each plant species in cm.

	Mean Difference	95.00% Confidence interval	P Value
Alfalfa			
<2.5 vs. 2.5–5	35.45	3.248 to 67.65	0.0234
<2.5 vs. 5–7.5	53.02	20.82 to 85.22	0.0001
<2.5 vs. 7.5–12.5	53.31	21.11 to 85.51	0.0001
<2.5 vs. >12.5	52.2	20.00 to 84.40	0.0002
2.5–5 vs. 5–7.5	17.57	−14.63 to 49.77	0.5547
2.5–5 vs. 7.5–12.5	17.86	−14.34 to 50.06	0.5387
2.5–5 vs. > 12.5	16.75	−15.45 to 48.95	0.6003
5–7.5 vs. 7.5–12.5	0.29	−31.91 to 32.49	>0.9999
5–7.5 vs. > 12.5	−0.82	−33.02 to 31.38	>0.9999
7.5–12.5 vs. > 12.5	−1.11	−33.31 to 31.09	>0.9999
Brome			
<2.5 vs. 2.5–5	49.72	17.52 to 81.92	0.0004
<2.5 vs. 5–7.5	60.06	27.86 to 92.26	<0.0001
<2.5 vs. 7.5–12.5	55.24	23.04 to 87.44	<0.0001
<2.5 vs. >12.5	64.08	31.88 to 96.28	<0.0001
2.5–5 vs. 5–7.5	10.34	−21.86 to 42.54	0.8992
2.5–5 vs. 7.5–12.5	5.52	−26.68 to 37.72	0.9893
2.5–5 vs. > 12.5	14.36	−17.84 to 46.56	0.7287
5–7.5 vs. 7.5–12.5	−4.82	−37.02 to 27.38	0.9936
5–7.5 vs. > 12.5	4.02	−28.18 to 36.22	0.9968
7.5–12.5 vs. > 12.5	8.84	−23.36 to 41.04	0.9406
Indiangrass			
<2.5 vs. 2.5–5	79.17	46.97 to 111.4	<0.0001
<2.5 vs. 5–7.5	78.7	46.50 to 110.9	<0.0001
<2.5 vs. 7.5–12.5	81.14	48.94 to 113.3	<0.0001
<2.5 vs. >12.5	84.24	52.04 to 116.4	<0.0001
2.5–5 vs. 5–7.5	−0.47	−32.67 to 31.73	>0.9999
2.5–5 vs. 7.5–12.5	1.97	−30.23 to 34.17	0.9998
2.5–5 vs. > 12.5	5.07	−27.13 to 37.27	0.9923
5–7.5 vs. 7.5–12.5	2.44	−29.76 to 34.64	0.9996
5–7.5 vs. > 12.5	5.54	−26.66 to 37.74	0.9892
7.5–12.5 vs. > 12.5	3.1	−29.10 to 35.30	0.9989
Kentucky Bluegrass			
<2.5 vs. 2.5–5	86.68	54.48 to 118.9	<0.0001
<2.5 vs. 5–7.5	89.98	57.78 to 122.2	<0.0001
<2.5 vs. 7.5–12.5	89.07	56.87 to 121.3	<0.0001
<2.5 vs. >12.5	91.43	59.23 to 123.6	<0.0001
2.5–5 vs. 5–7.5	3.3	−28.90 to 35.50	0.9985
2.5–5 vs. 7.5–12.5	2.39	−29.81 to 34.59	0.9996
2.5–5 vs. > 12.5	4.75	−27.45 to 36.95	0.994
5–7.5 vs. 7.5–12.5	−0.91	−33.11 to 31.29	>0.9999
5–7.5 vs. > 12.5	1.45	−30.75 to 33.65	>0.9999
7.5–12.5 vs. > 12.5	2.36	−29.84 to 34.56	0.9996
Orchardgrass			
<2.5 vs. 2.5–5	44.17	11.97 to 76.37	0.0022
<2.5 vs. 5–7.5	49.03	16.83 to 81.23	0.0005
<2.5 vs. 7.5–12.5	58.8	26.60 to 91.00	<0.0001
<2.5 vs. >12.5	43.24	11.04 to 75.44	0.0029
2.5–5 vs. 5–7.5	4.86	−27.34 to 37.06	0.9934
2.5–5 vs. 7.5–12.5	14.63	−17.57 to 46.83	0.7148
2.5–5 vs. > 12.5	−0.93	−33.13 to 31.27	>0.9999
5–7.5 vs. 7.5–12.5	9.77	−22.43 to 41.97	0.9165
5–7.5 vs. > 12.5	−5.79	−37.99 to 26.41	0.9872
7.5–12.5 vs. > 12.5	−15.56	−47.76 to 16.64	0.6655
Perennial Rye			
<2.5 vs. 2.5–5	44.38	12.18 to 76.58	0.0021
<2.5 vs. 5–7.5	51.24	19.04 to 83.44	0.0002
<2.5 vs. 7.5–12.5	51.22	19.02 to 83.42	0.0002
<2.5 vs. >12.5	48.51	16.31 to 80.71	0.0006
2.5–5 vs. 5–7.5	6.86	−25.34 to 39.06	0.976
2.5–5 vs. 7.5–12.5	6.84	−25.36 to 39.04	0.9763
2.5–5 vs. > 12.5	4.13	−28.07 to 36.33	0.9965
5–7.5 vs. 7.5–12.5	−0.02	−32.22 to 32.18	>0.9999
5–7.5 vs. > 12.5	−2.73	−34.93 to 29.47	0.9993
7.5–12.5 vs. > 12.5	−2.71	−34.91 to 29.49	0.9993
Timothy			
<2.5 vs. 2.5–5	53.52	21.32 to 85.72	0.0001
<2.5 vs. 5–7.5	55.02	22.82 to 87.22	<0.0001
<2.5 vs. 7.5–12.5	50.41	18.21 to 82.61	0.0003
<2.5 vs. >12.5	63.35	31.15 to 95.55	<0.0001
2.5–5 vs. 5–7.5	1.5	−30.70 to 33.70	>0.9999
2.5–5 vs. 7.5–12.5	−3.11	−35.31 to 29.09	0.9988
2.5–5 vs. > 12.5	9.83	−22.37 to 42.03	0.9148
5–7.5 vs. 7.5–12.5	−4.61	−36.81 to 27.59	0.9946
5–7.5 vs. > 12.5	8.33	−23.87 to 40.53	0.9518
7.5–12.5 vs. > 12.5	12.94	−19.26 to 45.14	0.7976
Sudangrass			
<2.5 vs. 2.5–5	82.29	50.09 to 114.5	<0.0001
<2.5 vs. 5–7.5	95.7	63.50 to 127.9	<0.0001
<2.5 vs. 7.5–12.5	96.35	64.15 to 128.6	<0.0001
<2.5 vs. >12.5	96.31	64.11 to 128.5	<0.0001
2.5–5 vs. 5–7.5	13.41	−18.79 to 45.61	0.7756
2.5–5 vs. 7.5–12.5	14.06	−18.14 to 46.26	0.7439
2.5–5 vs. > 12.5	14.02	−18.18 to 46.22	0.7459
5–7.5 vs. 7.5–12.5	0.65	−31.55 to 32.85	>0.9999
5–7.5 vs. > 12.5	0.61	−31.59 to 32.81	>0.9999
7.5–12.5 vs. > 12.5	−0.04	−32.24 to 32.16	>0.9999
Sweet Clover			
<2.5 vs. 2.5–5	56.29	24.09 to 88.49	<0.0001
<2.5 vs. 5–7.5	58.19	25.99 to 90.39	<0.0001
<2.5 vs. 7.5–12.5	61.62	29.42 to 93.82	<0.0001
<2.5 vs. >12.5	62.06	29.86 to 94.26	<0.0001
2.5–5 vs. 5–7.5	1.9	−30.30 to 34.10	0.9998
2.5–5 vs. 7.5–12.5	5.33	−26.87 to 37.53	0.9907
2.5–5 vs. > 12.5	5.77	−26.43 to 37.97	0.9874
5–7.5 vs. 7.5–12.5	3.43	−28.77 to 35.63	0.9983
5–7.5 vs. > 12.5	3.87	−28.33 to 36.07	0.9973
7.5–12.5 vs. > 12.5	0.44	−31.76 to 32.64	>0.9999
Red Clover			
<2.5 vs. 2.5–5	38.52	6.318 to 70.72	0.0107
<2.5 vs. 5–7.5	61.96	29.76 to 94.16	<0.0001
<2.5 vs. 7.5–12.5	56.82	24.62 to 89.02	<0.0001
<2.5 vs. >12.5	61.55	29.35 to 93.75	<0.0001
2.5–5 vs. 5–7.5	23.44	−8.762 to 55.64	0.263
2.5–5 vs. 7.5–12.5	18.3	−13.90 to 50.50	0.5144
2.5–5 vs. > 12.5	23.03	−9.172 to 55.23	0.2799
5–7.5 vs. 7.5–12.5	−5.14	−37.34 to 27.06	0.9919
5–7.5 vs. > 12.5	−0.41	−32.61 to 31.79	>0.9999
7.5–12.5 vs. > 12.5	4.73	−27.47 to 36.93	0.9941

### 3.3. Interspecies differences in L3 distribution

Our experiments did not reveal statistical differences among the ability of *T. colubriformis* to ascend upwards onto different plant species. Subjectively, Sudangrass and Kentucky bluegrass had very few larvae recovered above 2.5 cm (Figs 2 and 3). A relatively high amount of variation was seen in some plant species; we attributed this to plant population density which was difficult to control due to variable germination rates.

## 4. Discussion

Several studies have addressed the environmental impact of larval development in pasture forages. For example, abiotic factors such as temperature and moisture, are well known to contribute to larval success on pasture, and have been reviewed elsewhere [11,12]. In this study, we attempted to hold these variables constant under greenhouse conditions and instead focus on the plant species as the variable we aimed to interrogate. Plant species encounter a remarkable diverse collection of nematodes: some are pathogens of animals, some of plants, some are free-living. Broadly, we hypothesize that L3 have a maximum height they climb and that certain types of plants inhibit larval locomotion by producing chemical or physical defense systems. Although physical defense systems such as spines and thorns are well known to protect plants from arthropods or herbivores, less is known about their potential inhibition of nematodes [13]. On the other hand, secreted and chemical defense against nematodes are well-described [14]. For example, proteases of plants are known to degrade the cuticle of nematodes [15]. Plants also produce a plethora of phytotoxins that deter pests including tannins, terpenoids, flavonoids, and saponins [16–18]. Accordingly, feeding of plants with chemical defenses such as tannins has been explored as a strategy to mitigate strongyle parasites in herbivores [19–22]. While these innate defense mechanisms have been considered against primary pathogens of plants, they have not been examined with regard to how they might impact the life history of strongyle nematode parasites infecting vertebrates.

The primary consideration of our study was for applied practical grazing recommendations. We selected *T. colubriformis* since it can infect both sheep and calves and thus is often used as a model for anthelmintic activity against small intestinal strongyles. For several decades, grazing management has been advocated and considered for parasite control [23]. At the present time, industry publications are suggesting that grass isn’t grazed shorter than 4–5 inches (10–12 cm) practical grazing purposes. Despite finding these recommendations on webpages or university extension services [24], we could find relatively little primary data for these recommendations. In the present study, the majority of the larvae were found on the lowest 2.5 cm of the plant across the plant species tested. This agrees with many of the contemporary practical grazing recommendations. In our studies, less than 20% of the L3 were detected on the plants after 48 hours. Attempts to allow longer climbing periods in future studies may enhance the total percentage of L3 recovered. It is possible the remaining L3 responded to other factors and did not climb onto the forage. The practical implication of our results is the recommendation that overgrazing by livestock results in a greater risk of parasite ingestion. The limitation of this study is that our assays took place over 48 hours; longer assays may demonstrate the accumulation of larvae higher on the forage.

The long-term consideration of this study is to enhance our understanding of the behavior and fate of parasitic larvae in the environment. While many studies focus on the adult parasites, associated lesions, and treatment, fewer assess the factors that enable or disable strongyle larvae in the environment. Structural and phytochemical defenses against plant parasitic nematodes may also act to alter the ecology and success of vertebrate nematodes. For example, some plants secrete a physical barrier that is inhibitory for pests [25]. On the other hand, it is well known that plants produce anti-nematode phytochemicals. Some of the nematode specific compounds, known as ascarosides, activate plant defense systems against pathogens [26]. The ascarosides have been shown to affect dauer formation, sexual attraction, chemotaxis, and olfaction in nematodes [27].

Our study failed to highlight a significant inhibitory effect on *T. colubriformis* larvae, however, more nuanced controlled studies may shed light on pasture forages that are inhibitory to nematodes. For example, a limitation of our study is we did not account for plant biomass (e.g., cubic density of matter) which inherently varied among plant species. In addition, this study controlled for a constant number of larvae reaching each plant, and there may be factors involved in escaping the fecal pat that may have been neglected. This was because our primary focus was on grazing height. Likewise, variations in abiotic factors such as humidity and temperature were held constant, and these could affect plant phytochemical production. Our studies were conducted under stress-free conditions for the plants, that is, they did not have extraneous arthropod or stress from other pests. Indeed, plant phytochemicals are inducible [8], and the simultaneous presence of plant pathogens may induce defenses that are inhibitory to animal parasites. In future studies, long-term exposure to strongyle nematodes is needed since there may be a lag between the time the nematodes are introduced and the time the plant produces chemical defenses. This includes plant metabolites that affect nematode development, survival, and motility [28]. Conversely, nematodes modulate gene expression to evade plant defense systems and the duration of our studies was not likely to capture the time necessary for alterations in nematode gene transcription and translation [29]. Lastly, a limitation of this study was that while we used different pasture grasses and legumes, in theory there is an infinite number of cultivars/biotypes of these vegetative species. In this study we used seeds with population heterogeneity, in other words, those native to the region without specific breeding tactics. Subtle differences in plant genotype could produce more dramatic results. More broadly, in our experiments, we held greenhouse conditions constant in order to examine variables in plant species. Its possible that heat, humidity, or other abiotic factors could exacerbate differences among plant species when it comes to production of anti-nematode defense or climbing ability of larvae.

In summary, our study was supportive of general recommendations that overgrazing can result in an increased ingestion of *T. colubriformis*. Although it is assumed this is the trend for all strongyles, further studies are needed to assess other nematodes and the myriad of forages available throughout the world. The innate ability of different strongyles to ascend different types of plants requires further investigation. This study focused on forage species common in the Midwestern United States. In this study, we held abiotic factors static in an effort to discover plant variable that decreased nematode climbing. For the plant species studied and the duration of climbing, we were not able to demonstrate clear differences among L3 distribution or ability to ascend forage. Conducting experiments with longer climbing times, other plant species, or under abiotic conditions could reveal plant species that are discouraging or encouraging of infectious nematodes. This type of work will be valuable as we decrease reliance on chemical anthelmintics.

## Supporting information

S1 Table No differences were detected among height strata when comparing among different plant species. Tukey’s multiple comparison test; height above soil level are given for each plant species in cm. (DOCX)
