# Supplementary material for: Distribution of Trichostrongylus colubriformis on common pasture grasses and legumes from the Midwestern United States
Source: PLoS One. 2025 Apr 11;20(4):e0321367. doi: 10.1371/journal.pone.0321367 (PMC11990792; doi:10.1371/journal.pone.0321367)
Supplement: S1 Table — (DOCX) [file pone.0321367.s001.docx]

**Supplemental Table 1**. No differences were detected among height strata when comparing among different plant species. Tukey’s multiple comparison test; height above soil level are given for each plant species in cm.

|  | Mean Difference | 95.00% CI | P Value |
| --- | --- | --- | --- |
|  |  |  |  |
| <2.5 |  |  |  |
| Alfalfa vs. Brome | -6.95 | -44.46 to 30.56 | 0.9998 |
| Alfalfa vs. Indiangrass | -25.78 | -63.29 to 11.73 | 0.4476 |
| Alfalfa vs. Kentucky Bluegrass | -32.56 | -70.07 to 4.952 | 0.1477 |
| Alfalfa vs. Orchardgrass | -0.62 | -38.13 to 36.89 | >0.9999 |
| Alfalfa vs. Perennial Rye | -0.2 | -37.71 to 37.31 | >0.9999 |
| Alfalfa vs. Timothy | -5.59 | -43.10 to 31.92 | >0.9999 |
| Alfalfa vs. Sudangrass | -37.48 | -74.99 to 0.03178 | 0.0504 |
| Alfalfa vs. Sweet Clover | -9.12 | -46.63 to 28.39 | 0.9986 |
| Alfalfa vs. Red Clover | -5.06 | -42.57 to 32.45 | >0.9999 |
| Brome vs. Indiangrass | -18.83 | -56.34 to 18.68 | 0.833 |
| Brome vs. Kentucky Bluegrass | -25.61 | -63.12 to 11.90 | 0.4574 |
| Brome vs. Orchardgrass | 6.33 | -31.18 to 43.84 | >0.9999 |
| Brome vs. Perennial Rye | 6.75 | -30.76 to 44.26 | 0.9999 |
| Brome vs. Timothy | 1.36 | -36.15 to 38.87 | >0.9999 |
| Brome vs. Sudangrass | -30.53 | -68.04 to 6.982 | 0.2161 |
| Brome vs. Sweet Clover | -2.17 | -39.68 to 35.34 | >0.9999 |
| Brome vs. Red Clover | 1.89 | -35.62 to 39.40 | >0.9999 |
| Indiangrass vs. Kentucky Bluegrass | -6.78 | -44.29 to 30.73 | 0.9999 |
| Indiangrass vs. Orchardgrass | 25.16 | -12.35 to 62.67 | 0.4835 |
| Indiangrass vs. Perennial Rye | 25.58 | -11.93 to 63.09 | 0.4591 |
| Indiangrass vs. Timothy | 20.19 | -17.32 to 57.70 | 0.7688 |
| Indiangrass vs. Sudangrass | -11.7 | -49.21 to 25.81 | 0.9911 |
| Indiangrass vs. Sweet Clover | 16.66 | -20.85 to 54.17 | 0.9123 |
| Indiangrass vs. Red Clover | 20.72 | -16.79 to 58.23 | 0.7413 |
| Kentucky Bluegrass vs. Orchardgrass | 31.94 | -5.572 to 69.45 | 0.1666 |
| Kentucky Bluegrass vs. Perennial Rye | 32.36 | -5.152 to 69.87 | 0.1536 |
| Kentucky Bluegrass vs. Timothy | 26.97 | -10.54 to 64.48 | 0.3814 |
| Kentucky Bluegrass vs. Sudangrass | -4.92 | -42.43 to 32.59 | >0.9999 |
| Kentucky Bluegrass vs. Sweet Clover | 23.44 | -14.07 to 60.95 | 0.5857 |
| Kentucky Bluegrass vs. Red Clover | 27.5 | -10.01 to 65.01 | 0.3535 |
| Orchardgrass vs. Perennial Rye | 0.42 | -37.09 to 37.93 | >0.9999 |
| Orchardgrass vs. Timothy | -4.97 | -42.48 to 32.54 | >0.9999 |
| Orchardgrass vs. Sudangrass | -36.86 | -74.37 to 0.6518 | 0.0583 |
| Orchardgrass vs. Sweet Clover | -8.5 | -46.01 to 29.01 | 0.9992 |
| Orchardgrass vs. Red Clover | -4.44 | -41.95 to 33.07 | >0.9999 |
| Perennial Rye vs. Timothy | -5.39 | -42.90 to 32.12 | >0.9999 |
| Perennial Rye vs. Sudangrass | -37.28 | -74.79 to 0.2318 | 0.0528 |
| Perennial Rye vs. Sweet Clover | -8.92 | -46.43 to 28.59 | 0.9989 |
| Perennial Rye vs. Red Clover | -4.86 | -42.37 to 32.65 | >0.9999 |
| Timothy vs. Sudangrass | -31.89 | -69.40 to 5.622 | 0.1682 |
| Timothy vs. Sweet Clover | -3.53 | -41.04 to 33.98 | >0.9999 |
| Timothy vs. Red Clover | 0.53 | -36.98 to 38.04 | >0.9999 |
| Sudangrass vs. Sweet Clover | 28.36 | -9.152 to 65.87 | 0.3104 |
| Sudangrass vs. Red Clover | 32.42 | -5.092 to 69.93 | 0.1518 |
| Sweet Clover vs. Red Clover | 4.06 | -33.45 to 41.57 | >0.9999 |
|  |  |  |  |
| 2.5-5 |  |  |  |
| Alfalfa vs. Brome | 7.32 | -30.19 to 44.83 | 0.9998 |
| Alfalfa vs. Indiangrass | 17.94 | -19.57 to 55.45 | 0.8692 |
| Alfalfa vs. Kentucky Bluegrass | 18.67 | -18.84 to 56.18 | 0.8399 |
| Alfalfa vs. Orchardgrass | 8.1 | -29.41 to 45.61 | 0.9995 |
| Alfalfa vs. Perennial Rye | 8.73 | -28.78 to 46.24 | 0.999 |
| Alfalfa vs. Timothy | 12.48 | -25.03 to 49.99 | 0.9859 |
| Alfalfa vs. Sudangrass | 9.36 | -28.15 to 46.87 | 0.9983 |
| Alfalfa vs. Sweet Clover | 11.72 | -25.79 to 49.23 | 0.991 |
| Alfalfa vs. Red Clover | -1.99 | -39.50 to 35.52 | >0.9999 |
| Brome vs. Indiangrass | 10.62 | -26.89 to 48.13 | 0.9956 |
| Brome vs. Kentucky Bluegrass | 11.35 | -26.16 to 48.86 | 0.9928 |
| Brome vs. Orchardgrass | 0.78 | -36.73 to 38.29 | >0.9999 |
| Brome vs. Perennial Rye | 1.41 | -36.10 to 38.92 | >0.9999 |
| Brome vs. Timothy | 5.16 | -32.35 to 42.67 | >0.9999 |
| Brome vs. Sudangrass | 2.04 | -35.47 to 39.55 | >0.9999 |
| Brome vs. Sweet Clover | 4.4 | -33.11 to 41.91 | >0.9999 |
| Brome vs. Red Clover | -9.31 | -46.82 to 28.20 | 0.9984 |
| Indiangrass vs. Kentucky Bluegrass | 0.73 | -36.78 to 38.24 | >0.9999 |
| Indiangrass vs. Orchardgrass | -9.84 | -47.35 to 27.67 | 0.9975 |
| Indiangrass vs. Perennial Rye | -9.21 | -46.72 to 28.30 | 0.9985 |
| Indiangrass vs. Timothy | -5.46 | -42.97 to 32.05 | >0.9999 |
| Indiangrass vs. Sudangrass | -8.58 | -46.09 to 28.93 | 0.9992 |
| Indiangrass vs. Sweet Clover | -6.22 | -43.73 to 31.29 | >0.9999 |
| Indiangrass vs. Red Clover | -19.93 | -57.44 to 17.58 | 0.7819 |
| Kentucky Bluegrass vs. Orchardgrass | -10.57 | -48.08 to 26.94 | 0.9958 |
| Kentucky Bluegrass vs. Perennial Rye | -9.94 | -47.45 to 27.57 | 0.9973 |
| Kentucky Bluegrass vs. Timothy | -6.19 | -43.70 to 31.32 | >0.9999 |
| Kentucky Bluegrass vs. Sudangrass | -9.31 | -46.82 to 28.20 | 0.9984 |
| Kentucky Bluegrass vs. Sweet Clover | -6.95 | -44.46 to 30.56 | 0.9998 |
| Kentucky Bluegrass vs. Red Clover | -20.66 | -58.17 to 16.85 | 0.7445 |
| Orchardgrass vs. Perennial Rye | 0.63 | -36.88 to 38.14 | >0.9999 |
| Orchardgrass vs. Timothy | 4.38 | -33.13 to 41.89 | >0.9999 |
| Orchardgrass vs. Sudangrass | 1.26 | -36.25 to 38.77 | >0.9999 |
| Orchardgrass vs. Sweet Clover | 3.62 | -33.89 to 41.13 | >0.9999 |
| Orchardgrass vs. Red Clover | -10.09 | -47.60 to 27.42 | 0.997 |
| Perennial Rye vs. Timothy | 3.75 | -33.76 to 41.26 | >0.9999 |
| Perennial Rye vs. Sudangrass | 0.63 | -36.88 to 38.14 | >0.9999 |
| Perennial Rye vs. Sweet Clover | 2.99 | -34.52 to 40.50 | >0.9999 |
| Perennial Rye vs. Red Clover | -10.72 | -48.23 to 26.79 | 0.9953 |
| Timothy vs. Sudangrass | -3.12 | -40.63 to 34.39 | >0.9999 |
| Timothy vs. Sweet Clover | -0.76 | -38.27 to 36.75 | >0.9999 |
| Timothy vs. Red Clover | -14.47 | -51.98 to 23.04 | 0.9623 |
| Sudangrass vs. Sweet Clover | 2.36 | -35.15 to 39.87 | >0.9999 |
| Sudangrass vs. Red Clover | -11.35 | -48.86 to 26.16 | 0.9928 |
| Sweet Clover vs. Red Clover | -13.71 | -51.22 to 23.80 | 0.9734 |
|  |  |  |  |
| 5-7.5 |  |  |  |
| Alfalfa vs. Brome | 0.09 | -37.42 to 37.60 | >0.9999 |
| Alfalfa vs. Indiangrass | -0.1 | -37.61 to 37.41 | >0.9999 |
| Alfalfa vs. Kentucky Bluegrass | 4.4 | -33.11 to 41.91 | >0.9999 |
| Alfalfa vs. Orchardgrass | -4.61 | -42.12 to 32.90 | >0.9999 |
| Alfalfa vs. Perennial Rye | -1.98 | -39.49 to 35.53 | >0.9999 |
| Alfalfa vs. Timothy | -3.59 | -41.10 to 33.92 | >0.9999 |
| Alfalfa vs. Sudangrass | 5.2 | -32.31 to 42.71 | >0.9999 |
| Alfalfa vs. Sweet Clover | -3.95 | -41.46 to 33.56 | >0.9999 |
| Alfalfa vs. Red Clover | 3.88 | -33.63 to 41.39 | >0.9999 |
| Brome vs. Indiangrass | -0.19 | -37.70 to 37.32 | >0.9999 |
| Brome vs. Kentucky Bluegrass | 4.31 | -33.20 to 41.82 | >0.9999 |
| Brome vs. Orchardgrass | -4.7 | -42.21 to 32.81 | >0.9999 |
| Brome vs. Perennial Rye | -2.07 | -39.58 to 35.44 | >0.9999 |
| Brome vs. Timothy | -3.68 | -41.19 to 33.83 | >0.9999 |
| Brome vs. Sudangrass | 5.11 | -32.40 to 42.62 | >0.9999 |
| Brome vs. Sweet Clover | -4.04 | -41.55 to 33.47 | >0.9999 |
| Brome vs. Red Clover | 3.79 | -33.72 to 41.30 | >0.9999 |
| Indiangrass vs. Kentucky Bluegrass | 4.5 | -33.01 to 42.01 | >0.9999 |
| Indiangrass vs. Orchardgrass | -4.51 | -42.02 to 33.00 | >0.9999 |
| Indiangrass vs. Perennial Rye | -1.88 | -39.39 to 35.63 | >0.9999 |
| Indiangrass vs. Timothy | -3.49 | -41.00 to 34.02 | >0.9999 |
| Indiangrass vs. Sudangrass | 5.3 | -32.21 to 42.81 | >0.9999 |
| Indiangrass vs. Sweet Clover | -3.85 | -41.36 to 33.66 | >0.9999 |
| Indiangrass vs. Red Clover | 3.98 | -33.53 to 41.49 | >0.9999 |
| Kentucky Bluegrass vs. Orchardgrass | -9.01 | -46.52 to 28.50 | 0.9988 |
| Kentucky Bluegrass vs. Perennial Rye | -6.38 | -43.89 to 31.13 | >0.9999 |
| Kentucky Bluegrass vs. Timothy | -7.99 | -45.50 to 29.52 | 0.9995 |
| Kentucky Bluegrass vs. Sudangrass | 0.8 | -36.71 to 38.31 | >0.9999 |
| Kentucky Bluegrass vs. Sweet Clover | -8.35 | -45.86 to 29.16 | 0.9993 |
| Kentucky Bluegrass vs. Red Clover | -0.52 | -38.03 to 36.99 | >0.9999 |
| Orchardgrass vs. Perennial Rye | 2.63 | -34.88 to 40.14 | >0.9999 |
| Orchardgrass vs. Timothy | 1.02 | -36.49 to 38.53 | >0.9999 |
| Orchardgrass vs. Sudangrass | 9.81 | -27.70 to 47.32 | 0.9976 |
| Orchardgrass vs. Sweet Clover | 0.66 | -36.85 to 38.17 | >0.9999 |
| Orchardgrass vs. Red Clover | 8.49 | -29.02 to 46.00 | 0.9992 |
| Perennial Rye vs. Timothy | -1.61 | -39.12 to 35.90 | >0.9999 |
| Perennial Rye vs. Sudangrass | 7.18 | -30.33 to 44.69 | 0.9998 |
| Perennial Rye vs. Sweet Clover | -1.97 | -39.48 to 35.54 | >0.9999 |
| Perennial Rye vs. Red Clover | 5.86 | -31.65 to 43.37 | >0.9999 |
| Timothy vs. Sudangrass | 8.79 | -28.72 to 46.30 | 0.999 |
| Timothy vs. Sweet Clover | -0.36 | -37.87 to 37.15 | >0.9999 |
| Timothy vs. Red Clover | 7.47 | -30.04 to 44.98 | 0.9997 |
| Sudangrass vs. Sweet Clover | -9.15 | -46.66 to 28.36 | 0.9986 |
| Sudangrass vs. Red Clover | -1.32 | -38.83 to 36.19 | >0.9999 |
| Sweet Clover vs. Red Clover | 7.83 | -29.68 to 45.34 | 0.9996 |
|  |  |  |  |
| 7.5-12.5 |  |  |  |
| Alfalfa vs. Brome | -5.02 | -42.53 to 32.49 | >0.9999 |
| Alfalfa vs. Indiangrass | 2.05 | -35.46 to 39.56 | >0.9999 |
| Alfalfa vs. Kentucky Bluegrass | 3.2 | -34.31 to 40.71 | >0.9999 |
| Alfalfa vs. Orchardgrass | 4.87 | -32.64 to 42.38 | >0.9999 |
| Alfalfa vs. Perennial Rye | -2.29 | -39.80 to 35.22 | >0.9999 |
| Alfalfa vs. Timothy | -8.49 | -46.00 to 29.02 | 0.9992 |
| Alfalfa vs. Sudangrass | 5.56 | -31.95 to 43.07 | >0.9999 |
| Alfalfa vs. Sweet Clover | -0.81 | -38.32 to 36.70 | >0.9999 |
| Alfalfa vs. Red Clover | -1.55 | -39.06 to 35.96 | >0.9999 |
| Brome vs. Indiangrass | 7.07 | -30.44 to 44.58 | 0.9998 |
| Brome vs. Kentucky Bluegrass | 8.22 | -29.29 to 45.73 | 0.9994 |
| Brome vs. Orchardgrass | 9.89 | -27.62 to 47.40 | 0.9974 |
| Brome vs. Perennial Rye | 2.73 | -34.78 to 40.24 | >0.9999 |
| Brome vs. Timothy | -3.47 | -40.98 to 34.04 | >0.9999 |
| Brome vs. Sudangrass | 10.58 | -26.93 to 48.09 | 0.9957 |
| Brome vs. Sweet Clover | 4.21 | -33.30 to 41.72 | >0.9999 |
| Brome vs. Red Clover | 3.47 | -34.04 to 40.98 | >0.9999 |
| Indiangrass vs. Kentucky Bluegrass | 1.15 | -36.36 to 38.66 | >0.9999 |
| Indiangrass vs. Orchardgrass | 2.82 | -34.69 to 40.33 | >0.9999 |
| Indiangrass vs. Perennial Rye | -4.34 | -41.85 to 33.17 | >0.9999 |
| Indiangrass vs. Timothy | -10.54 | -48.05 to 26.97 | 0.9958 |
| Indiangrass vs. Sudangrass | 3.51 | -34.00 to 41.02 | >0.9999 |
| Indiangrass vs. Sweet Clover | -2.86 | -40.37 to 34.65 | >0.9999 |
| Indiangrass vs. Red Clover | -3.6 | -41.11 to 33.91 | >0.9999 |
| Kentucky Bluegrass vs. Orchardgrass | 1.67 | -35.84 to 39.18 | >0.9999 |
| Kentucky Bluegrass vs. Perennial Rye | -5.49 | -43.00 to 32.02 | >0.9999 |
| Kentucky Bluegrass vs. Timothy | -11.69 | -49.20 to 25.82 | 0.9911 |
| Kentucky Bluegrass vs. Sudangrass | 2.36 | -35.15 to 39.87 | >0.9999 |
| Kentucky Bluegrass vs. Sweet Clover | -4.01 | -41.52 to 33.50 | >0.9999 |
| Kentucky Bluegrass vs. Red Clover | -4.75 | -42.26 to 32.76 | >0.9999 |
| Orchardgrass vs. Perennial Rye | -7.16 | -44.67 to 30.35 | 0.9998 |
| Orchardgrass vs. Timothy | -13.36 | -50.87 to 24.15 | 0.9776 |
| Orchardgrass vs. Sudangrass | 0.69 | -36.82 to 38.20 | >0.9999 |
| Orchardgrass vs. Sweet Clover | -5.68 | -43.19 to 31.83 | >0.9999 |
| Orchardgrass vs. Red Clover | -6.42 | -43.93 to 31.09 | >0.9999 |
| Perennial Rye vs. Timothy | -6.2 | -43.71 to 31.31 | >0.9999 |
| Perennial Rye vs. Sudangrass | 7.85 | -29.66 to 45.36 | 0.9996 |
| Perennial Rye vs. Sweet Clover | 1.48 | -36.03 to 38.99 | >0.9999 |
| Perennial Rye vs. Red Clover | 0.74 | -36.77 to 38.25 | >0.9999 |
| Timothy vs. Sudangrass | 14.05 | -23.46 to 51.56 | 0.9688 |
| Timothy vs. Sweet Clover | 7.68 | -29.83 to 45.19 | 0.9997 |
| Timothy vs. Red Clover | 6.94 | -30.57 to 44.45 | 0.9999 |
| Sudangrass vs. Sweet Clover | -6.37 | -43.88 to 31.14 | >0.9999 |
| Sudangrass vs. Red Clover | -7.11 | -44.62 to 30.40 | 0.9998 |
| Sweet Clover vs. Red Clover | -0.74 | -38.25 to 36.77 | >0.9999 |
|  |  |  |  |
| >12.5 |  |  |  |
| Alfalfa vs. Brome | 4.93 | -32.58 to 42.44 | >0.9999 |
| Alfalfa vs. Indiangrass | 6.26 | -31.25 to 43.77 | >0.9999 |
| Alfalfa vs. Kentucky Bluegrass | 6.67 | -30.84 to 44.18 | 0.9999 |
| Alfalfa vs. Orchardgrass | -9.58 | -47.09 to 27.93 | 0.998 |
| Alfalfa vs. Perennial Rye | -3.89 | -41.40 to 33.62 | >0.9999 |
| Alfalfa vs. Timothy | 5.56 | -31.95 to 43.07 | >0.9999 |
| Alfalfa vs. Sudangrass | 6.63 | -30.88 to 44.14 | 0.9999 |
| Alfalfa vs. Sweet Clover | 0.74 | -36.77 to 38.25 | >0.9999 |
| Alfalfa vs. Red Clover | 4.29 | -33.22 to 41.80 | >0.9999 |
| Brome vs. Indiangrass | 1.33 | -36.18 to 38.84 | >0.9999 |
| Brome vs. Kentucky Bluegrass | 1.74 | -35.77 to 39.25 | >0.9999 |
| Brome vs. Orchardgrass | -14.51 | -52.02 to 23.00 | 0.9616 |
| Brome vs. Perennial Rye | -8.82 | -46.33 to 28.69 | 0.999 |
| Brome vs. Timothy | 0.63 | -36.88 to 38.14 | >0.9999 |
| Brome vs. Sudangrass | 1.7 | -35.81 to 39.21 | >0.9999 |
| Brome vs. Sweet Clover | -4.19 | -41.70 to 33.32 | >0.9999 |
| Brome vs. Red Clover | -0.64 | -38.15 to 36.87 | >0.9999 |
| Indiangrass vs. Kentucky Bluegrass | 0.41 | -37.10 to 37.92 | >0.9999 |
| Indiangrass vs. Orchardgrass | -15.84 | -53.35 to 21.67 | 0.9344 |
| Indiangrass vs. Perennial Rye | -10.15 | -47.66 to 27.36 | 0.9969 |
| Indiangrass vs. Timothy | -0.7 | -38.21 to 36.81 | >0.9999 |
| Indiangrass vs. Sudangrass | 0.37 | -37.14 to 37.88 | >0.9999 |
| Indiangrass vs. Sweet Clover | -5.52 | -43.03 to 31.99 | >0.9999 |
| Indiangrass vs. Red Clover | -1.97 | -39.48 to 35.54 | >0.9999 |
| Kentucky Bluegrass vs. Orchardgrass | -16.25 | -53.76 to 21.26 | 0.9239 |
| Kentucky Bluegrass vs. Perennial Rye | -10.56 | -48.07 to 26.95 | 0.9958 |
| Kentucky Bluegrass vs. Timothy | -1.11 | -38.62 to 36.40 | >0.9999 |
| Kentucky Bluegrass vs. Sudangrass | -0.04 | -37.55 to 37.47 | >0.9999 |
| Kentucky Bluegrass vs. Sweet Clover | -5.93 | -43.44 to 31.58 | >0.9999 |
| Kentucky Bluegrass vs. Red Clover | -2.38 | -39.89 to 35.13 | >0.9999 |
| Orchardgrass vs. Perennial Rye | 5.69 | -31.82 to 43.20 | >0.9999 |
| Orchardgrass vs. Timothy | 15.14 | -22.37 to 52.65 | 0.95 |
| Orchardgrass vs. Sudangrass | 16.21 | -21.30 to 53.72 | 0.925 |
| Orchardgrass vs. Sweet Clover | 10.32 | -27.19 to 47.83 | 0.9965 |
| Orchardgrass vs. Red Clover | 13.87 | -23.64 to 51.38 | 0.9713 |
| Perennial Rye vs. Timothy | 9.45 | -28.06 to 46.96 | 0.9982 |
| Perennial Rye vs. Sudangrass | 10.52 | -26.99 to 48.03 | 0.9959 |
| Perennial Rye vs. Sweet Clover | 4.63 | -32.88 to 42.14 | >0.9999 |
| Perennial Rye vs. Red Clover | 8.18 | -29.33 to 45.69 | 0.9994 |
| Timothy vs. Sudangrass | 1.07 | -36.44 to 38.58 | >0.9999 |
| Timothy vs. Sweet Clover | -4.82 | -42.33 to 32.69 | >0.9999 |
| Timothy vs. Red Clover | -1.27 | -38.78 to 36.24 | >0.9999 |
| Sudangrass vs. Sweet Clover | -5.89 | -43.40 to 31.62 | >0.9999 |
| Sudangrass vs. Red Clover | -2.34 | -39.85 to 35.17 | >0.9999 |
| Sweet Clover vs. Red Clover | 3.55 | -33.96 to 41.06 | >0.9999 |
